# Emirates Society of Radiation Oncology (ESRO): Advancing Cancer Care in the UAE

**DOI:** 10.7759/cureus.80500

**Published:** 2025-03-12

**Authors:** Nandan M Shanbhag, Abdulrahman Bin Sumaida, Aly A Razek, Yashaswini Shivashankara, Khalifa AlKaabi, Khalid Balaraj

**Affiliations:** 1 Radiation Oncology/Palliative Care, Tawam Hospital, Al Ain, ARE; 2 Oncology/Radiation Oncology, Tawam Hospital, Al Ain, ARE; 3 Radiation Oncology, Gulf International Cancer Centre, Abu Dhabi, ARE; 4 Radiation Oncology, Mediclinic Middle East, Abu Dhabi, ARE; 5 Internal Medicine, College of Medicine and Health Sciences, United Arab Emirates University, Al Ain, ARE; 6 Radiation Oncology, Tawam Hospital, Al Ain, ARE

**Keywords:** cancer care, emirates society of radiation oncology, esro, medical education, oncology research, policy advocacy, quality assurance, radiation oncology, radiotherapy, uae

## Abstract

Radiation oncology plays a crucial role in cancer treatment, with more than half of all cancer patients requiring radiotherapy at some stage. Despite rapid advancements in oncology services in the United Arab Emirates (UAE), the country has lacked a dedicated professional society for radiation oncology until now. The Emirates Society of Radiation Oncology (ESRO) was established in 2025 to address critical gaps in education, research, clinical practice, and policy advocacy within the field. This editorial highlights the rationale for ESRO’s formation, including workforce training deficits, limited research collaboration, and the absence of unified clinical guidelines. ESRO aims to enhance professional development, foster multicenter research, advocate for evidence-based policy reforms, and standardize radiation oncology practices across the UAE. Through these efforts, ESRO seeks to elevate the quality of radiotherapy services, improve patient outcomes, and position the UAE as a key contributor to global advancements in radiation oncology. We call on all stakeholders, including oncologists, medical physicists, radiation therapists, policymakers, and healthcare institutions, to collaborate in realizing this vision for a stronger and more integrated radiation oncology community in the region.

## Editorial

Introduction

Radiation therapy is a cornerstone of modern cancer treatment, with more than half of all cancer patients requiring radiotherapy at some point in their care [1]. In the United Arab Emirates (UAE), the cancer burden has been steadily rising, with 4,633 new cancer cases recorded in 2019, and cancer is now the third leading cause of death nationally, accounting for approximately 13% of all deaths [2]. Over the past few decades, the UAE has significantly expanded its oncology infrastructure, growing from a single radiotherapy center in the 1980s to at least ten centers providing radiation therapy services by 2027. Advanced technologies such as intensity-modulated and image-guided radiotherapy, CyberKnife radiosurgery, and the first MR-Linac in the Middle East have been introduced, reflecting the country’s commitment to cutting-edge cancer care [3-5].

Despite this progress, until now, there has been no dedicated professional society for radiation oncology in the UAE to unify and lead these efforts. Most radiation oncologists and allied professionals have been organizing under general oncology groups without a focused forum for radiation therapy [6]. This gap has limited opportunities for the specialized exchange of knowledge, the development of tailored guidelines, and advocacy specific to radiotherapy. In response to this need, the Emirates Society of Radiation Oncology (ESRO) was established in 2025 as the first specialty society for radiation oncology in the UAE. This editorial discusses the current challenges that prompted ESRO’s formation, the society’s objectives, and its potential impact on the future of cancer care in the UAE and beyond.

Current challenges in radiation oncology in the UAE

Several key challenges in education, research, and clinical practice have highlighted the need for a dedicated radiation oncology society in the UAE.

Workforce Training and Education Gaps

There is a lack of local specialized training programs for critical staff such as medical physicists and radiation therapists. Most of these professionals in the UAE have had to obtain their qualifications abroad. Similarly, radiation oncologists have traditionally been trained overseas, as no comprehensive domestic residency program in radiation oncology has existed. This gap in training infrastructure risks workforce shortages and highlights the need for structured educational initiatives [7].

Fragmented Research and Limited Academic Output

Cancer research in the UAE has lagged behind the rapid improvements in clinical care. Historically, very few oncology clinical trials have been conducted in the country, and research activity has been impeded by barriers at healthcare, institutional, and regulatory levels. In 2022, a review found that the UAE had far fewer oncology trials registered compared to neighboring countries, highlighting an urgent need for better research networks [8]. The absence of a unifying body meant that collaborative multicenter research and data sharing in radiation oncology were minimal.

Lack of a Unified Professional Platform

Without an official radiation oncology society, practitioners had no single platform to regularly convene, share expertise, or develop consensus guidelines specific to the UAE’s context. The previous lack of a professional organization limited the ability to standardize practice protocols across different centers.

Policy and Quality Assurance Challenges

As radiotherapy centers quickly proliferated, ensuring consistent quality and safety is paramount. Variations in technology and practice standards between centers can lead to inconsistencies in patient care. Additionally, while the rapid growth in radiotherapy capacity has improved access, it raises the need for coordinated planning to avoid duplication of services and to maintain high treatment volumes per center, which are linked to better quality outcomes. Without a collective voice, radiation oncology professionals have faced challenges in advocating for policy changes, such as updated reimbursement models or national guidelines, that could address these issues.

These challenges provided a clear rationale for forming ESRO. By addressing educational gaps, research fragmentation, and the lack of unified standards, a dedicated society can help elevate radiation oncology to meet international benchmarks and ensure that advancements in technology translate into optimal patient outcomes in the UAE.

Objectives of ESRO

ESRO’s mission is to advance the field of radiation oncology in the UAE through a multi-pronged approach. The society’s key objectives include the following.

Professional Development and Education

ESRO will serve as a platform for the continuous medical education of radiation oncologists, medical physicists, dosimetrists, and radiation therapists. This involves organizing workshops, symposiums, and conferences to update members on the latest evidence and techniques. A top priority is to develop local training pathways, such as advocating for accredited residency and fellowship programs in radiation oncology and establishing training programs for allied professionals. Through mentorship and educational resources, ESRO aims to nurture the next generation of Emirati radiation oncology experts.

Research Collaboration and Innovation

A core role of ESRO is to foster collaborative research across the UAE’s cancer centers. The society will encourage multicenter clinical trials, prospective registries, and data-sharing initiatives focused on radiotherapy outcomes. By uniting stakeholders, ESRO can help overcome the fragmented research landscape and low clinical trial accrual that have hampered oncology research in the past. ESRO aims to focus on radiotherapy-specific research questions, promote investigator-initiated studies, and partner with international research groups. The ultimate goal is to generate homegrown research that addresses local needs and contributes to global scientific knowledge.

Clinical Practice Guidelines and Quality Standards

As a professional body, ESRO will coordinate the development of consensus guidelines and recommendations to standardize radiotherapy practice across the UAE. These may cover protocols for common cancers, safety checks, dosimetry standards, and the adoption of new technologies. By consulting national experts and referencing international best practices, the society can issue guidance that helps harmonize care. Furthermore, ESRO can work closely with health regulators to establish a national quality assurance framework for radiation oncology. The society’s involvement in policy dialogue can ensure that equipment procurement, technology upgrades, and workforce staffing in radiotherapy align with evidence-based standards and the country’s actual needs.

Public Awareness

The society will also engage in public outreach to raise awareness about the safety and benefits of radiotherapy, helping dispel myths and encouraging patients’ confidence in modern radiation treatments.

Improving Patient Care and Outcomes

All of ESRO’s activities ultimately aim to improve patient care. By elevating education, research, and clinical standards, the society will help ensure that cancer patients across the Emirates receive state-of-the-art radiotherapy delivered safely and effectively. ESRO can implement tumor boards or case discussion forums for complex cases, enabling collective expert input on patient management. It will also promote multidisciplinary collaboration, integrating radiation oncology more closely with surgical and medical oncology teams for coordinated decision-making. Through its programs, ESRO strives for tangible impacts: shorter waiting times for radiotherapy, more precise treatment with fewer side effects, and better cancer control and survival rates. In essence, the society will channel the collective expertise of its members to continuously improve the quality of radiation therapy available to patients in the UAE.

Future impact and global contribution

The formation of ESRO is poised to significantly shape the future of radiation oncology in the UAE. In the short term, improved coordination and communication among the country’s radiotherapy centers can be expected. A society-driven collaborative network will facilitate the rapid sharing of clinical experiences and new research findings, benefiting all member institutions. This collaborative spirit is especially crucial as the field becomes increasingly subspecialized. No single center can master all emerging techniques in isolation, but together, practitioners can pool their expertise for the common good. Additionally, as ESRO promotes unified guidelines, practice variations between centers are likely to diminish, leading to more consistent care regardless of treatment location.

In the medium term, ESRO’s influence on training and research will start bearing fruit. Enhanced educational opportunities will attract more medical graduates into the field, easing workforce constraints. On the research front, an ESRO-facilitated increase in clinical trials and studies will generate data tailored to the region’s multiethnic population. The UAE’s diverse patient demographics present a unique opportunity for research insights, as patients hail from various Middle Eastern, Asian, and Western backgrounds. By tapping into this diversity through multicenter research, ESRO can help produce findings that are relevant not just locally but globally. For instance, outcomes data or toxicity profiles gleaned in the UAE could inform treatment approaches in other countries with similar demographics.

ESRO can also play a pivotal role in guiding technology adoption and innovation in the UAE’s radiotherapy sector. The society provides a forum to evaluate new treatment modalities, such as proton therapy or MR-guided radiotherapy, and advise on their implementation. A collective approach ensures that expensive technologies are introduced rationally and complemented by proper training. Moreover, ESRO can encourage innovation by supporting pilot projects in areas such as artificial intelligence (AI) for treatment planning and quality assurance. Incorporating AI and machine learning into radiotherapy has been highlighted as a promising avenue to improve efficiency and standardize care. With ESRO’s backing, UAE centers could collaborate on deploying novel tools, such as using AI to automate aspects of contouring or plan verification, thereby positioning the UAE at the forefront of radiotherapy innovation in the region.

Beyond national borders, ESRO will serve as a bridge linking the UAE’s radiation oncology community with the international oncology sphere. The society can establish partnerships with global organizations such as the European Society for Radiotherapy and Oncology (ESTRO) and the American Society for Radiation Oncology (ASTRO). Such collaborations may include joint meetings, training exchanges, or participation in international guideline development. With a formal body in place, the UAE can more effectively contribute to and benefit from global advances. In time, ESRO’s model could inspire greater regional cooperation, perhaps by collaborating with neighboring Gulf countries on research or forming a Middle East radiation oncology consortium to address common challenges, similar to emerging Middle East radiation oncology groups. Ultimately, ESRO amplifies the UAE’s voice in global cancer control discussions and helps ensure that the country is not just a consumer of international best practices but also a contributor to worldwide progress in radiation oncology.

Conclusion

The establishment of the ESRO marks a milestone in the UAE’s healthcare landscape, uniting radiation oncology professionals to advance education, research, and clinical excellence. Its success depends on collaboration, as radiation oncologists, physicists, therapists, administrators, and policymakers must join forces to elevate cancer care. We urge institutions to support ESRO’s mission by fostering participation, research, and best practices. As the UAE embraces world-class healthcare innovation, ESRO will ensure that every cancer patient receives cutting-edge radiotherapy. This is a call to action: through unity, knowledge-sharing, and leadership, ESRO will shape the future of radiation oncology in the UAE and beyond.
